# A Few Cases of True Pelvic Cellulitis—A Plea for More Thorough Pelvic Surgery1Read at the Twentieth Annual Meeting of the American Gynecological Society, Baltimore, May, 1895.

**Published:** 1895-07

**Authors:** Ely Van De Warker

**Affiliations:** 404 Fayette Park; Syracuse, N. Y.


### A FEW CASES OF TRUE PELVIC CELLULITIS—A PLEA FOR MORE THOROUGH PELVIC SURGERY.[1]

By ELY VAN DE WARKER, Syracuse, N. Y.

It is not many years since, under the stimulus of what was then called progress, that the term "pelvic cellulitis" was denied a place as a condition of disease. It was regarded as a phantom, the product of half knowledge, if not of ignorance.

After holding absolute sway as the leading factor in pathology of the female pelvis, according to the best authority of the time, it was deposed and actually driven out of respectable literature by the pelvic surgeon. By the fingers of the laparatomist it was demonstrated beyond all doubt that what had been frequently regarded as an inflammation of the cellular connective tissue, was but an adhesion of near parts due to an inflammatory exudate, or a disease of the tubes and ovaries while the cellular structure remained intact. This condition was found so frequently that the existence of collections of pus in the pelvic connective tissue was regarded as rarely, if ever seen, and that such collections of pus were in the Fallopian tubes, or in peritoneal spaces shut in by adhesive exudate, or designated by the collective and uncertain name of "pus sacs."

All scientific men must admit that this was a true advance, and that a vague and erroneous theory of pelvic inflammation was overthrown. It is positively a fact that the term "pelvic cellulitis" was used in a most unscientific and even ignorant way. By it was explained all forms of pelvic adhesions, exudates and pelvic fixations and masses not evidently due to neoplasms. This was cleared up by the pelvic surgeon. It was the revolt of the man of facts against the despotism of the man of theory. But to one who used facts as a mental search-light after truth it was but the exchange of one form of despotism for another, more arrogant and aggressive than the first.

---

1. Read at the Twentieth Annual Meeting of the American Gynecological Society, Baltimore, May, 1895.

The pelvic, tubal or ovarian extirpator was intolerant of the very term of cellular inflammation. Peritoneal and tubal disease simply took the place of cellular disease as the one and ever-present agent of pelvic inflammation. Here the salpingotomist, after all his triumphs, rested and became, like the theorist whom he displaced, an obstacle to advancement. Established error gave way before the logic of the facts that he had demonstrated. Had he stopped there the advance would have been borne without a regret ; but he did more. He obliterated the very idea of cellular inflammation as a cause of pelvic disease, and brought the term under such contempt that one who had regard for his reputation as a scientific man and a safe diagnostician among the body of the profession hesitated to admit its possibility.

After the performance of untold thousands of pelvic sections for the cure of pelvic inflammatory conditions, a vast number of which must have been useless and harmful, the more observing among this group of surgeons became aware of the fact that many of their patients were not cured, and were even made worse. Among them were numerous cases where enormous tubal accumulations clearly indicated the operation. Masses of adhesions reformed and uterine fixation recurred, or was not relieved.

After vaginal hysterectomy became well established and a comparatively simple operation, the mysterious obstacle in the way of success was supposed to be the uterus, and the more advanced surgeon proposed in cases where tubal and ovarian disease demanded extirpation to remove the uterus also as the really offending and now useless organ.

The facts of the pelvic surgeon had also lapsed into theory as narrow as the one that he had displaced and equally at fault as an explanation of the total phenomena of pelvic inflammation. There are evident indications that the crude operation of tubal and ovarian extirpation, as a general operation in pelvic inflammation, is becoming obsolete and that the future line of surgical relief will come from the direction of the hysterectomist.

The reaction began to revive the old idea of pelvic cellulitis. It was the old theory, but seen in a new light. It was a crystallization out of the mass of crude logic and obliquely observed facts of the old authors. But it is now a well-defined and scientific term. We know what it is as well as what it is not. Limited in this way it is a positive advance in our knowledge of pelvic inflammation, and broadens the etiological factors of pelvic disease. * * * *

These cases are selected out of many as having an illustrative value upon several points involved in this paper, and not in any sense as proof of the condition known as pelvic cellulitis, the existence of which, I take it for granted, no fair-minded and careful observer has the least doubt. One of the misfortunes of surgical specialism is to develop a tendency to mental restriction. One cannot live, think and act in a narrow purview for years without limiting one's mental perspective. It is a matter like this that appears to have entered into this question. The pelvic surgeon seems to have ignored the possibility of any wider area of pelvic inflammation than that which attacks the peritoneum, the tubes or the ovaries.

This was the status of pelvic pathology through a period of splendid surgery and of which I believe we are beginning to see the decline. Here and there a pelvic surgeon, as some of those who make a specialty of exploring the pelvis through an abdominal incision wish to be called, became awake to the fact that there was another pelvic morbid entity than peritoneal adhesions, saculated tubes, cystic ovaries and pus sacs, and that notwithstanding the thorough and aseptic removal of these conditions—and no one with any experience can doubt the propriety of the operation—the patient did not get well. The pain, the neuroses, the pelvic fixations and the disability continued in full force. Several surgeons who candidly admitted these negative results in published articles, and recommend uterine extirpation, which I do not hesitate to admit was a proper conclusion, reached it, in my opinion, upon false premises. They accused the most inoffensive organ in the female pelvis unless it be the seat of malignancy, or of neoplasms—namely, the uterus—of being the offender. It is true that it had become a superfluous organ, and also true that some prompt recoveries followed its extirpation after tubal and ovarian operations. I believe that the failure to cure after the primary operation was due to an associated pelvic cellular inflammation and not to the fact that the uterus was left intact.

In my public and private practice I have had abundant material, have operated liberally and have been often disappointed. Some of the conditions found were very misleading. Enormously distended tubes, ovaries incarcerated in masses of exudate and adhesion, omental and intestinal and uterine adhesions appeared to account for all the condition of the patient, though removal of the offending conditions and prompt recovery from the operation failed to relieve the patient. My experience in Case III. was repeated several times and always with good results. But this method was tedious, lacked precision, exposed the patient to the danger of secondary infection, and was too limited in its action.

I have always been found in the ranks of the conservative pelvic surgeons, but I became convinced that we did not go far enough and that the uterus ought to be removed—not because it was diseased, but by the removal of the organ we opened up the cellular pelvic spaces and secured the necessary drainage to relieve the cellulitis. Hysterectomy, in my opinion, affords the only route to this area of intrapelvic inflammation.

I am also convinced that it is hysterectomy of a certain kind. Preferably it is through the vagina, but it is not the operation by ligation, but by the forceps or clamp. It makes a wide difference whether all the spaces are occluded and surfaces are brought into contact by sutures and inclusive ligatures, or are left open to free drainage by the removal of the forceps, which secures this result by the retracting issues. The French method is now developing along this line, and if we are correctly informed, with brilliant results.

If I am asked, Ought this method to be practised in all cases, I emphatically answer no. We may have pelvic peritonitis with its resulting evils without associated pelvic cellulitis. We may have many forms of tubal disease imperatively demanding operation unattended with inflammation of the pelvic cellular spaces, and we may have the latter without the former.

What I am contending for is a scientific recognition of the various forms of inflammation, and I believe that we may reach a reasonable differential diagnosis ; not in all cases, for I have a profound respect for the difficulties of pelvic diagnosis by palpation. But in very considerable number we may come to a safe conclusion. I have a simple diagnostic sign, and when I find it I feel very sure of the presence of cellular inflammation. In this tissue, pelvic or elsewhere, inflammation may extend by continuity into any part made up of like histological elements. We have inflammation in the broad ligament extending into the iliac fossa laterally, and downward through connective tissue spaces into the vaginal septum, and through the pubo-sacral areolar process to the lateral surfaces of the vagina. (Savage.) In these locations the infiltrated cellular spaces can be brought up between the finger in the rectum and the thumb in the vagina as thickened indurated and doughy masses. This condition may extend downward an inch or more. It may extend to the lateral surfaces of the vagina, disappearing to the touch above the limits of the passage and shows on palpation as a slightly elastic mass firmly fixed to the pelvic wall, smooth and glistening, the vaginal rugæ obliterated. I have observed that these lateral extensions reach downward farther than those situated in the recto-vaginal wall, reaching in some instances nearly to the vestibule. This condition is a true cellulitis and indicates and is concurrent with cellulitis higher up in the pelvic space; but is not associated with pelvic peritonitis or salpingitis or oöphoritis.

While this vaginal cellulitis has been described, I have not seen it referred to as an index of the character of the primary pelvic inflammation. Unless existing in a very marked degree, it cannot positively be detected by vaginal examination alone, but by combined vaginal and rectal palpation. We must remember, however, that there is no reason why pelvic peritonitis and cellulitis may not coëxist. Having this possibility in view, the symptom I have just described could prove the intercurrence of cellular inflammation.

When this is well established I believe that thorough surgical treatment requires hysterectomy as well as the older operation of removal of tubes and ovaries.

404 Fayette Park.

